# Hemostasis during the early stages of trauma: comparison with disseminated intravascular coagulation

**DOI:** 10.1186/cc13816

**Published:** 2014-04-03

**Authors:** Akiko Oshiro, Yuichiro Yanagida, Satoshi Gando, Naomi Henzan, Isao Takahashi, Hiroshi Makise

**Affiliations:** 1 Emergency and Critical Care Center, Teine Keijinkai Hospital, Sapporo, Japan; 2 Division of Acute and Critical Care Medicine, Department of Anesthesiology and Critical Care Medicine, Hokkaido University Graduate School of Medicine, N15W7, Kita-ku, Sapporo 060-8638, Japan; 3 Emergency and Critical Care Center, Sapporo City General Hospital, Sapporo, Japan

## Abstract

**Introduction:**

We tested two hypotheses that disseminated intravascular coagulation (DIC) and acute coagulopathy of trauma-shock (ACOTS) in the early phase of trauma are similar disease entities and that the DIC score on admission can be used to predict the prognosis of patients with coagulopathy of trauma.

**Methods:**

We conducted a retrospective study of 562 trauma patients, including 338 patients whose data were obtained immediately after admission to the emergency department. We collected serial data for the platelet counts, global markers of coagulation and fibrinolysis, and antithrombin levels. DIC was diagnosed according to the Japanese Association for Acute Medicine (JAAM) DIC scoring system, and ACOTS was defined as a prothrombin-time ratio of >1.2.

**Results:**

The higher levels of fibrin/fibrinogen degradation products (FDP) and D-dimer and greater FDP/D-dimer ratios in the DIC patients suggested DIC with the fibrinolytic phenotype. The DIC patients with the fibrinolytic phenotype exhibited persistently lower platelet counts and fibrinogen levels, increased prothrombin time ratios, higher FDP and D-dimer levels, and lower antithrombin levels compared with the non-DIC patients on arrival to the emergency department and during the early stage of trauma. Almost all ACOTS patients met the criteria for a diagnosis of DIC; therefore, the same changes were observed in the platelet counts, global markers of coagulation and fibrinolysis, and antithrombin levels as noted in the DIC patients. The JAAM DIC score obtained immediately after arrival to the emergency department was an independent predictor of massive transfusion and death due to trauma and correlated with the amount of blood transfused.

**Conclusions:**

Patients who develop DIC with the fibrinolytic phenotype during the early stage of trauma exhibit consumption coagulopathy associated with increased fibrin(ogen)olysis and lower levels of antithrombin. The same is true in patients with ACOTS. The JAAM DIC score can be used to predict the prognosis of patients with coagulopathy of trauma.

## Introduction

The coagulopathy of trauma is a syndrome characterized by a nonsurgical oozing-type of bleeding from mucosal regions, serosal surfaces and wounds, and vascular-access sites that is distinct from simple massive bleeding and is caused by multiple factors, such as hypothermia, acidosis, hemodilution, hemorrhagic shock, and serious trauma itself. For more than four decades, trauma- and shock-induced disseminated intravascular coagulation (DIC) with the fibrinolytic phenotype has been believed to be the primary cause of coagulopathy of trauma [1,2]. Persistently lower platelet counts and fibrinogen levels, more-prolonged prothrombin times, increased fibrinogen and fibrin degradation product (FDP) levels, and low levels of proteins that control coagulation (antithrombin) and fibrinolysis (α2-plasmin inhibitor) have been repeatedly confirmed in DIC patients during the early to late phases of trauma, irrespective of the use of massive platelet concentrate and fresh frozen plasma (FFP) transfusions [2,3].

Two decades after the discovery of trauma- and shock-induced DIC, Brohi *et al*. [4] and MacLoad *et al*. [5] reconfirmed that trauma itself is the main cause of coagulopathy of trauma [2]. Based on this discovery, another concept of coagulopathy of trauma called acute coagulopathy of trauma-shock (ACOTS) was established as a distinct disease entity distinct from DIC [6,7]. In this concept, ACOTS, not DIC, is considered to be the primary pathophysiological mechanism of coagulopathy of trauma. The authors asserted that no evidence implicates the process of DIC in the development of coagulopathy of trauma [6-8]. Legitimate rebuttals of these concepts have reviewed the similarities and differences in DIC and ACOTS and reconfirmed the appropriateness of the DIC concept [9,10]. Until recently, almost all studies on ACOTS have discussed its pathophysiology based on single-point sampling of blood or single-point measurement of the parameters of viscoelastic devices [9,10]. Therefore, data regarding serial changes in the platelet counts and global markers of coagulation and fibrinolysis in patients with ACOTS are lacking.

In the present study, to test the hypothesis that similarities exist between DIC with the fibrinolytic phenotype and ACOTS, we measured platelet counts, global markers of coagulation and fibrinolysis, and antithrombin levels in trauma patients immediately after arrival to the emergency department and during the early phase of trauma. Another hypothesis was that DIC diagnostic criteria consisting of various markers of coagulation and fibrinolysis can be used to predict the prognosis of trauma patients with coagulopathy of trauma.

Recently, coagulopathy of trauma has been termed trauma-induced coagulopathy, whereas the term ACOTS has been replaced with acute traumatic coagulopathy. However, the present study uses the original terminology, coagulopathy of trauma and ACOTS as a representative nomenclature for the various terminologies derived from ACOTS without a clear definition.

## Materials and methods

### Patient selection and data collection

This retrospective study was conducted at three tertiary critical care centers with the approval of the institutional review board of each hospital (Institutional Review Board of Hokkaido University Hospital for Clinical Research; Ethics Committee of Sapporo City General Hospital; and Ethics Committee of Teine Keijinkai Hospital). The need for informed consent was waived because of the anonymous, observational, and retrospective nature of the study. Severe trauma patients, defined as Injury Severity Score (ISS) ≥9 (at least one abbreviated injury scale ≥3), were enrolled in the study. Trauma patients with cardiac arrest or those resuscitated from cardiac arrest, as well as individuals receiving anticoagulant therapy and those having known clotting disorders, such as hematopoietic malignancies or severe liver cirrhosis, were excluded. A systematic review of the computer-based medical records of these patients was retrospectively conducted to obtain baseline characteristics, levels of coagulation and fibrinolytic markers, and DIC-related variables. The data for these variables were obtained on day 0 (on admission) and on days 1 and 2. Patient data that could be collected immediately on arrival to the emergency department were obtained at four time points within 24 hours after arrival: time point 0, immediately after arrival at the emergency department; time point 1, 4 to 8 hours after arrival; time point 2, 8 to 16 hours after arrival; and time point 3, 16 to 24 hours after arrival.

### Definitions and diagnosis

The severity of illness was evaluated according to the Acute Physiology and Chronic Health Evaluation (APACHE) II score within 24 hours after the admission [11]. Organ failure was assessed by using the Sequential Organ Failure Assessment (SOFA) score [12]. The systemic inflammatory response syndrome (SIRS) was defined according to criteria of the American College of Chest Physicians/Society of Critical Care Medicine consensus conference [13]. Multiple organ dysfunction syndrome (MODS) was defined as a SOFA score ≥12 [12]. Massive bleeding (requiring transfusion) was defined as the total amount of packed red blood cells (PRBCs) and FFP ≥2,000 ml (approximately 50% of the circulating blood volume of an average Japanese individual) transfused for 24 hours from the time of initial presentation to the emergency department. DIC diagnosis was made based on the Japanese Association for Acute Medicine (JAAM) DIC diagnosis criteria [14]. The overt DIC score, determined according to the International Society on Thrombosis and Haemostasis (ISTH), was also calculated [15]. The FDP level was used as the fibrin-related marker in the ISTH criteria. No increase, moderate increase, and strong increase were defined as an FDP level of <9, 10 to 24, and >25 mg/L, respectively. The DIC scores were calculated by using laboratory tests performed at the same time points. When the total score was ≥4 and ≥5, the JAAM and ISTH overt DIC diagnosis was established, respectively. Patients who did not undergo all of the required laboratory tests were diagnosed as having JAAM and ISTH overt DIC if the sum of the available data was ≥4 and ≥5.

In the present study, ISTH DIC was defined as that the JAAM DIC patients simultaneously met the ISTH overt DIC criteria. ACOTS was defined as a prothrombin time ratio of >1.2 based on the announcement that a prothrombin time ratio of >1.2 should be adopted as the clinically relevant definition of ACOTS by the multinational ACOTS group [16]. This group declared this definition as appropriate for future use and indicated that they expect the use of this definition to increase significantly the reported incidence of ACOTS. The outcome measure was all-cause hospital mortality, including death in the emergency department.

### Measurement methods and treatment

Prospective blood sampling was performed on admission to the emergency department and daily thereafter, as part of a routine clinical and laboratory workup using established standard laboratory techniques at each hospital. Platelet count, prothrombin time ratio, and the levels of fibrinogen, FDP, D-dimer, and antithrombin were collected. The platelet counts (XE-5000; Sysmex, Tokyo, Japan), and levels of fibrinogen (Thrombocheck Fib; Sysmex), FDP (NanopiaP-FDP, Sekisuimedical, Tokyo), and D-dimer (Nanopia D-dimer; Sekisuimedical) were measured by using the same kits in three hospitals. The prothrombin time (Tromborel S; Siemens, and CoagpiaN, Sekisuimedical) and antithrombin levels (Testtime ATIII, Sekisuimedical; Lsystem ATIII, Sysmex; Antithrombin S, Siemens) were measured by using different kits in three hospitals. In addition, blood-gas analyses were used for lactate measurement. Treatment selection was at the discretion of each critical care center; however, standard strategies for treating trauma were applied based on the Japan Advanced Trauma Evaluation and Care guidelines established by the Japanese Association for the Surgery of Trauma.

### Statistical analysis

All data are expressed as the mean ± standard deviation. The IBM SPSS 21.0 for MAC OSX software package (IBM Japan, Tokyo) was used for all statistical-calculation analyses. Comparisons between the two groups were made by using the Mann–Whitney *U* test and either the χ^2^ test or Fisher Exact test if necessary. To compare three groups, the Kruskal-Wallis test was applied. The relations between the measured variables and mortality or massive bleeding were analyzed by stepwise logistic regression analysis (the backward stepwise method based on likelihood) with the use of death or massive bleeding as dependent variables. The results were reported as the odds ratios and 95% confidence intervals (CIs). A multiple regression with stepwise method was applied to predict the amount of transfusion. A value of *P* < 0.05 was considered to be statistically significant.

## Results

### Baseline patient characteristics

In total, 562 patients were included in the present study. The baseline characteristics of these patients are presented in Table 1. The JAAM DIC patients, especially those who simultaneously met the ISTH overt DIC criteria, had more serious injuries and higher APACHE II and SOFA scores, therefore exhibiting poor outcomes associated with MODS. These results demonstrated that the prognosis of the trauma patients deteriorated in accordance with increasing abnormalities of coagulation and fibrinolysis (that is, the severity of DIC). Higher FDP and D-dimer levels as well as greater FDP/D-dimer ratios in the DIC patients were observed as a result of increased fibrin(ogen)olysis, suggesting the presence of DIC with the fibrinolytic phenotype.

**Table 1:** Baseline characteristics of all 562 patients included in the study

	**Non-DIC**	**JAAM DIC**		***P *** **value**
	**(*n*** **= 257)**	**ISTH (−) (*n*** **= 210)**	**ISTH (+) (*n*** **= 95)**	
Age (years)	48 ± 20	49 ± 23	49 ± 25	0.682
Sex (male/female)	64/193	76/134	43/52	0.001
Injury Severity Score	20.7 ± 11.5	25.7 ± 13.0	35.3 ± 13.0	0.000
APACHE II score	13.9 ± 7.2	19.7 ± 8.2	26.1 ± 8.1	0.000
SOFA score	5.0 ± 2.2	6.5 ± 3.3	10.2 ± 3.6	0.000
SIRS criteria	2.8 ± 0.8	3.4 ± 0.7	3.0 ± 0.9	0.000
JAAM DIC score (day 0)	1.5 ± 1.0	4.7 ± 1.0	6.7 ± 1.3	0.000
ISTH DIC score (day 0)	1.2 ± 1.2	3.3 ± 0.6	5.9 ± 1.0	0.000
MODS (yes/no)	2/255	22/188	41/54	0.000
Outcome (survived/died)	248/9	174/36	54/41	0.000
FDP/D-dimer ratio	1.6 ± 0.9	1.8 ± 0.7	4.3 ± 19.8	0.000
Lactate (mM)	3.2 ± 1.9	4.9 ± 3.2	8.2 ± 5.1	0.000

APACHE, Acute Physiology and Chronic Health Evaluation; DIC, disseminated intravascular coagulation; FDP, fibrin fibrinogen degradation products; FFP, fresh frozen plasma; ISTH, International Society on Thrombosis and Haemostasis; JAAM, Japanese Association for Acute Medicine; MODS, multiple organ dysfunction syndrome; PRBCs, packed red blood cells; SIRS, systemic inflammatory response syndrome; SOFA, Sequential Organ Failure Assessment. ISTH (−) (+), JAAM DIC patients who did not meet the ISTH overt DIC criteria and those who simultaneously met the ISTH overt DIC criteria, respectively. *P* value, Kruskal-Wallis test.

Of the 562 patients, the data for 338 patients were collected immediately after admission to the emergency department. The baseline characteristics of these patients are presented in Table 2. In addition to having the same characteristics as the DIC patients shown in Table 1, the DIC patients received higher levels of transfused platelet concentrate, PRBCs, and FFP than the non-DIC patients within 24 hours after arrival to the emergency department. As a result, the DIC patients frequently met the criteria for a massive transfusion.

**Table 2:** Baseline characteristics of the 338 patients whose data were collected immediately after arrival in the emergency department

	**Non-DIC**	**JAAM DIC**		***P *** **value**
	**(*n*** **= 137)**	**ISTH (−) (*n*** **= 131)**	**ISTH (+) (*n*** **= 70)**	
Age (years)	48 ± 20	47 ± 23	47 ± 24	0.862
Sex (male/female)	40/97	46/85	32/38	0.062
Injury severity score	22.1 ± 12.1	27.9 ± 13.4	37.5 ± 12.2	0.000
APACHE II score	15.6 ± 7.5	21.6 ± 8.4	26.4 ± 8.3	0.000
SOFA score	5.6 ± 2.9	7.4 ± 3.6	10.9 ± 3.6	0.000
SIRS criteria	2.9 ± 0.9	3.3 ± 0.7	3.0 ± 0.9	0.000
JAAM DIC score	1.6 ± 0.9	4.7 ± 0.9	6.3 ± 1.3	0.000
ISTH DIC score	1.3 ± 1.2	3.2 ± 0.7	6.0 ± 0.9	0.000
MODS (yes/no)	4/133	26/105	31/39	0.000
Outcome (survived/died)	127/10	100/31	36/34	0.000
FDP/D-dimer ratio	1.8 ± 1.1	1.9 ± 0.7	5.3 ± 23.8	0.002
FFP/PRBC transfusion ratio	0.7 ± 0.4	1.0 ± 0.9	1.1 ± 0.9	0.073
Lactate (m*M*)	3.6 ± 2.6	4.8 ± 3.5	7.8 ± 4.9	0.000
Platelet concentrate (U)	3.4 ± 9.1	4.5 ± 10.2	23.0 ± 24.8	0.000
Packed red blood cells (ml)	247 ± 708	666 ± 1,442	2,655 ± 3,180	0.000
Fresh frozen plasma (ml)	159 ± 431	575 ± 1,101	2,275 ± 2,586	0.000
Massive transfusion (yes/no)	11/126	29/102	48/22	0.000

Abbreviations, see Table 1. *P* value, Kruscal-Wallis test. The JAAM and ISTH DIC scores were calculated by using the data obtained immediately after arrival in the emergency department (time point 0 in Figure 3). The amounts of platelet concentrate, packed red blood cells, and fresh frozen plasma indicate the total amount of volume transfused within 24 hours after the arrival in the emergency department.

**Figure 1:**
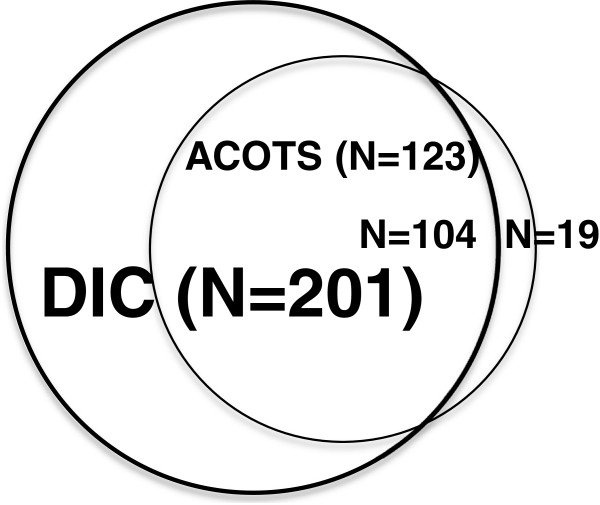
**Relations between DIC (bold circle, *n*** **= 201) and ACOTS (open circle, *n*** **= 123).** Almost all ACOTS patients (*n* = 104) were included as DIC patients. Only 19 patients with ACOTS did not meet the DIC criteria.

We found that 174 of the total 562 patients and 123 of the 338 patients met the criteria for ACOTS. The baseline characteristics of the ACOTS patients are presented in Table 3, which shows that the patients exhibited similarities to the characteristics of DIC. Almost all of the ACOTS patients whose data were obtained immediately after arrival in the emergency department were diagnosed as having JAAM DIC (104 of 123; 84.6%); however, 19 of the 123 ACOTS patients did not fulfill the JAAM DIC criteria. Figure 1 shows the relation between ACOTS (123 of 338) and JAAM DIC patients (201 of 338) diagnosed immediately on admission to the emergency department.

**Table 3:** Baseline characteristics of the patients with acute coagulopathy of trauma-shock (ACOTS)

	**ACOTS**^**a**^	**ACOTS**^**b**^	**ACOTS**^**c**^
	**Day 0 (*n*** **= 174)**	**On admission (*n*** **= 123)**	**On admission (*n*** **= 19)**
Age (years)	46 ± 23	46 ± 23	52 ± 18
Sex (male/female)	64/114	74/49	13/6
Injury Severity Score	31.0 ± 13.7	32.6 ± 13.6	20.9 ± 11.3
APACHE II score	22.5 ± 8.9	23.5 ± 8.9	19.4 ± 8.8
SOFA score	9.1 ± 3.9	9.8 ± 3.8	8.4 ± 3.0
SIRS criteria	3.1 ± 0.9	3.1 ± 0.9	3.0 ± 1.0
JAAM DIC score	5.6 ± 1.9	5.2 ± 1.8	2.1 ± 0.5
ISTH DIC score	4.6 ± 1.7	4.5 ± 1.9	1.7 ± 1.2
MODS (yes/no)	52/122	41/63	2/17
Outcome (survived/died)	122/52	75/48	16/3
Prothrombin time ratio	1.7 ± 1.1	1.7 ± 0.9	1.4 ± 0.3
Platelet concentrate (U)	-	14.0 ± 21.7	5.7 ± 13.5
Packed red blood cell (ml)	-	1,715 ± 2,609	382 ± 547
Fresh frozen plasma (ml)	-	1,422 ± 2,074	195 ± 284

Abbreviations, see Table 1. ^a^Of the total 562 patients, the day 0 data for 174 patients who met the ACOTS criteria are shown. ^b^Of the 338 patients whose data were obtained immediately after admission, 123 met the ACOTS criteria, and their data on admission are presented. ^c^The data for 19 patients who met the ACOTS criteria but not the JAAM DIC criteria are shown. Of the 123 ACOTS patients, 19 did not meet the JAAM DIC criteria.

### Changes in the platelet counts and global markers of coagulation and fibrinolysis

**Figure 2:**
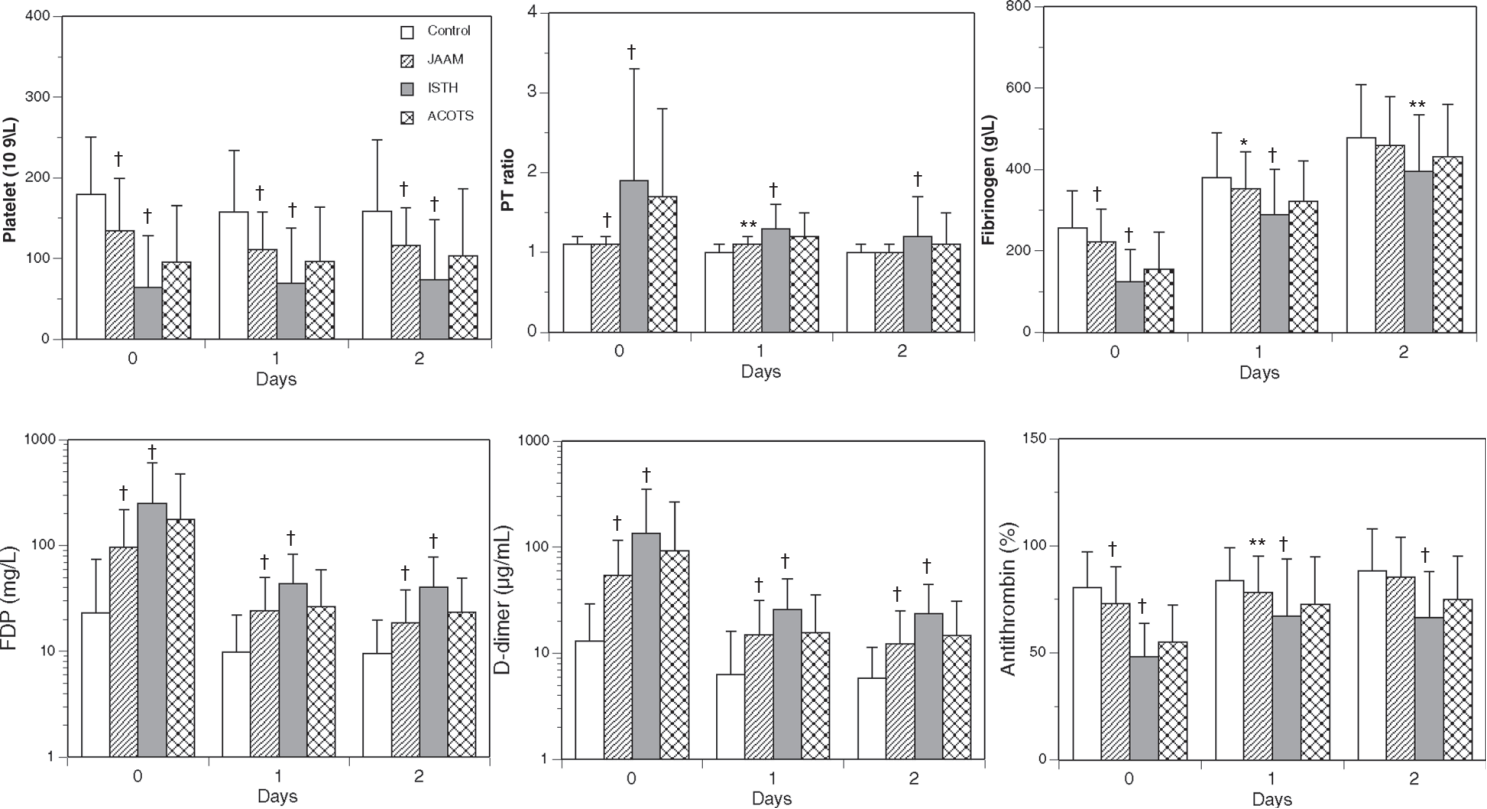
**The bar graphs show the changes in the platelet counts, prothrombin time ratio (PT ratio), and levels of fibrinogen, FDP, D-dimer, and antithrombin in the total 562 patients.** ACOTS, patients who met the ACOTS diagnostic criteria; control, patients who did not meet the JAAM DIC criteria; ISTH, patients who simultaneously met both the JAAM and ISTH DIC criteria; JAAM, patients who met the JAAM DIC criteria. The ACOTS patients overlapped with control, JAAM, and ISTH patients. *P < 0.05; **P < 0.01; †*P* < 0.001 versus control.

**Figure 3:**
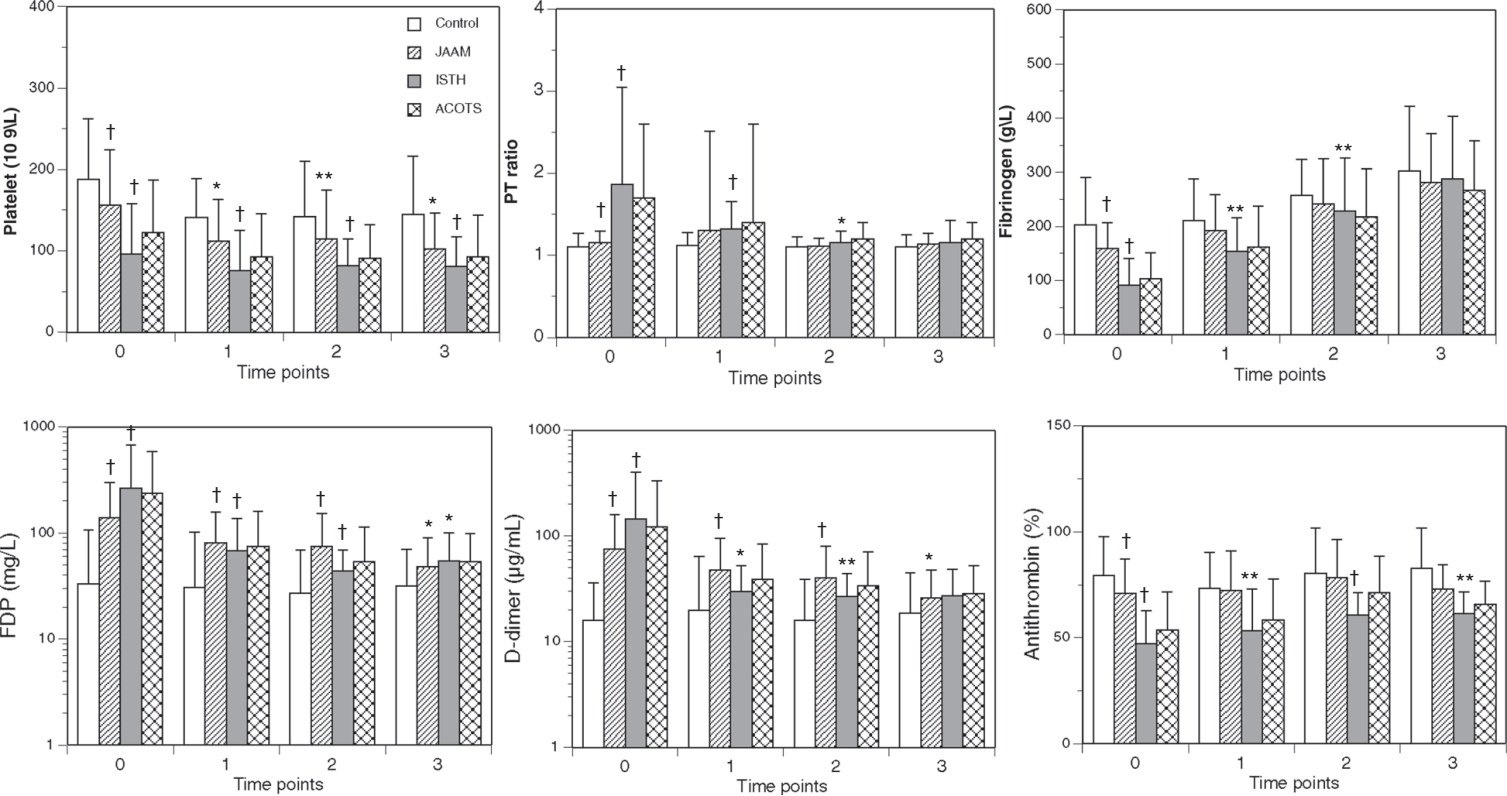
**The bar graphs show the changes in the platelet counts, prothrombin-time ratio (PT ratio), and levels of fibrinogen, FDP, D-dimer, and antithrombin in the 338 patients for whom data were collected immediately after admission in the emergency department.** ACOTS, patients who met the ACOTS diagnostic criteria; control, patients who did not meet the JAAM DIC criteria; ISTH, patients who simultaneously met both the JAAM and ISTH DIC criteria; JAAM, patients who met the JAAM DIC criteria. The ACOTS patients overlapped with the control, JAAM, and ISTH patients. For time points 0 to 3, refer to the Methods section in detail. *P < 0.05; **P < 0.01; †*P* < 0.001 versus control.

Figure 2 shows the changes in markers in all 562 patients. Consistent with the results of previous studies on DIC after trauma, persistently lower platelet counts and fibrinogen levels, higher prothrombin time ratios, higher levels of FDP and D-dimer, and lower levels of antithrombin were observed in the DIC patients compared with those observed in the non-DIC patients from day 0 to day 2 [3]. These changes were more pronounced in the patients who simultaneously met the ISTH overt DIC criteria. Figure 3 shows the changes in markers of 338 patients whose data were collected immediately after arrival in the emergency department. The same time courses were observed in the platelet counts, coagulation and fibrinolysis markers, and antithrombin levels as those noted in the 562 patients during the first 24 hours after arrival in the emergency department.

Figures 2 and 3 also show the changes in the platelet counts, global markers of coagulation and fibrinolysis, and antithrombin levels in the patients with ACOTS. Because of the large overlap of the patients with DIC and ACOTS, a statistical analysis was not applied to the patients with ACOTS. However, Figures 2 and 3 clearly demonstrate that the changes in the platelet counts, global markers of coagulation and fibrinolysis, and antithrombin levels in the patients with ACOTS coincided with those of the DIC patients. The details of the number of patients in Figures 2 and 3 are presented in Additional file 1.

### Outcome prediction and need for massive transfusion

Data obtained immediately after arrival in the emergency department are critical for predicting the prognosis of severely injured trauma patients with massive bleeding and hemorrhagic shock. Therefore, the data of the 338 patients for whom data were available immediately after admission were used to predict the prognoses of the patients in this study. Table 4 shows that both the JAAM DIC score and the fibrinogen level on admission could be used to predict patient death and the need for a massive transfusion. Table 5 indicates that the amounts of transfused platelet concentrate and PRBC and FFP correlated strongly with the JAAM DIC score and fibrinogen level on admission.

**Table 4:** Stepwise logistic regression analysis for predicting death and massive transfusion in the 338 patients

	**Odds ratio**	***P *** **value**	**95% confidence interval**
Death			
Age	1.038	0.000	1.019–1.057
Platelet counts	1.110	0.001	1.045–1.179
Prothrombin time ratio	4.718	0.007	1.529–14.557
Fibrinogen	0.990	0.021	0.982–0.999
FDP	1.005	0.001	1.002–1.007
JAAM DIC score	3.129	0.035	1.084–9.033
Massive transfusion			
Fibrinogen	0.991	0.001	0.986–0.997
JAAM DIC score	4.607	0.001	1.935–10.972

Abbreviations, see Table 1. Independent variables, age, sex, platelet count, prothrombin time ratio, fibrinogen, FDP, and JAAM DIC score on admission.

**Table 5:** Multiple regression analysis using the stepwise method for predicting the transfusion of platelet concentrate and the total amount of packed red blood cells (PRBCs) and fresh frozen plasma (FFP)

	**B (SE)**	**β**	***P *** **value**	**95% CI**	***R***^**2**^	**ANOVA**
Platelet concentrate					0.207	0.000
Platelet count	−0.27 (0.13)	−0.13	0.045	−0.524 to −0.007		
Prothrombin time ratio	4.0 (1.8)	0.16	0.025	0.509 to 7.401		
FDP	−0.02 (0.005)	−0.21	0.002	−0.024 to −0.005		
JAAM DIC score	2.8 (0.5)	0.35	0.000	1.749 to 3.757		
Transfusion (PRBCs and FFP)					0.169	0.000
JAAM DIC score	701 (92)	0.41	0.000	518.4 to 884.3		

B, partial regression coefficient; SE, standard error; β, standard partial regression coefficient; CI, confidence interval; *R*^2^, coefficient of determination; ANOVA, analysis of variance. Independent variables: platelet count, prothrombin time ratio, the fibrinogen level, the FDP levels, and the JAAM DIC score on admission.

## Discussion

The higher levels of FDP and D-dimer and higher FDP/D-dimer ratios observed in the present patients indicate that the DIC diagnosed in this study was the fibrinolytic phenotype [2,10]. Our study demonstrates that patients who develop DIC with the fibrinolytic phenotype immediately after trauma exhibit consumption coagulopathy associated with increased fibrin(ogen)olysis and lower levels of antithrombin [1,3]. The same findings were true in the patients with ACOTS.

In Japan, emergency medical teams are not permitted to infuse and/or transfuse any fluids or blood products in trauma patients during transportation. The standard practice is to draw blood immediately after the patient arrives in the emergency department to analyze parameters of coagulation and fibrinolysis and other laboratory data. Therefore, the data obtained at time point 0 in Figure 3 were not influenced by either hemodilution or hypothermia. A possibility exists that hemodilution could have affected the changes in the measured parameters, especially the platelet counts and levels of fibrinogen and antithrombin at time points 1 to 3. However, the guidelines for transfusion therapy and the proper use of blood products published by the Japanese Ministry of Health Labor and Welfare in 2005 clearly state that complications of consumption coagulopathy caused by DIC should always be examined in trauma patients, and if consumption coagulopathy exists, trauma specialists should consider transfusing a sufficient amount of FFP [2]. The high FFP/PRBC transfusion ratio observed in the DIC patients indicates the application of these guidelines, which may have prevented the effect of hemodilution.

The use of sampling time points is important for assessing coagulopathy during the early stage of trauma [9,10]. The hemostatic changes that occur after trauma are rapidly dynamic processes; therefore, obtaining serial measurements of multiple markers of coagulation and fibrinolysis is essential. Almost all ACOTS studies using only single-point sampling of blood on arrival in the emergency department, especially those that lack a control cohort, should elicit caution when interpreting the results with respect to hemostasis or pathologic coagulopathy [9,10]. To our knowledge, this is the first study showing that the serial changes in platelet counts, global markers of coagulation and fibrinolysis, and antithrombin levels in patients with ACOTS during the early stage of trauma were equal to those of DIC patients. Both the JAAM DIC and ISTH overt DIC diagnostic criteria include the prothrombin time ratio or prothrombin time in their scoring systems, which easily explains the overlap between DIC and ACOTS patients observed in the present study, as well as in our previous study [17].

ACOTS is an incomprehensible disease entity with almost the same diagnostic criteria as DIC, but a clearly different pathophysiology. Thrombin generation increases in the circulation in DIC patients but is inhibited by activated protein C in patients with ACOTS, which is the reason for their bleeding tendency [7-10]. Liberal transfusion of FFP-containing protein C, a source of activated protein C, would theoretically inhibit thrombin formation and aggravate ACOTS. FFP also contains anticoagulants against thrombin, such as antithrombin and tissue-factor pathway inhibitor, as well as coagulation factors; therefore, FFP transfusion is recommended for DIC patients to control thrombin generation and to treat consumption coagulopathy [2,9,10]. On the contrary, the antithrombin levels should be kept as low as possible to enhance the thrombin formation in ACOTS patients. Therefore, FFP transfusion is reasonably contraindicated in trauma patients with ACOTS if its basic principles were correct [2,9,10,18,19].

Dunbar *et al*. [20,21] elegantly demonstrated marked systemic thrombin generation caused by circulating procoagulants, such as tissue factor and microparticles, and a reduced ability to localize hemostasis at the wound site due to the loss of antithrombin in patients with ACOTS. The antithrombin levels in both the DIC and ACOTS patients observed in the present and previous studies were lower than those in the control subjects [17,18]. A multiple regression analysis clearly indicated that a lower antithrombin level is the major determinant of a systemic increase in thrombin generation and activity in DIC patients [17]. It should therefore be noted that the coagulation-control pathways induced by antithrombin are insufficient to anchor thrombin at the injured site after trauma in patients with both DIC and ACOTS. These results obtained from multiple studies theoretically support the use of FFP transfusion to correct consumption coagulopathy in DIC patients [22] and reveal contradictions in the pathophysiology of ACOTS [9,10].

By using data obtained immediately after arrival in the emergency department and data readily available worldwide, we analyzed the prognosis of trauma patients in the present study. The results showed that the JAAM DIC score and fibrinogen level on admission can be used to predict both the need for massive transfusions within 24 hours after presentation in the emergency room and in-hospital patient death. In addition, the FDP level was found to be an independent predictor of patient death, suggesting the important role of fibrin(ogen)olysis in DIC with the fibrinolytic phenotype [23]. Multivariate analysis suggested that the total amount of transfused platelet concentrate, PRBC, and FFP correlated significantly with the JAAM DIC score, platelet count, prothrombin time ratio, and FDP level. However, the coefficients of determination (*R*^2^) were relatively low at 0.207 for platelet concentrate and 0.169 for PRBC and FFP (Table 5). These results indicate that other factors are involved in determining the transfusion volume during the early stage of trauma.

### Limitations

The retrospective nature of this study, missing data from days 1 and 2 and time points 1 through 3 and the use of different reagents and kits to measure the markers levels between the hospitals may limit interpretation of our results. In addition, we did not examine microvascular fibrin thrombosis, the gold standard for the diagnosis of “DIC with the thrombotic phenotype.” However, it has long been recognized that demonstrating fibrin thrombosis is dependent on the time elapsed to autopsy and that microvascular thrombosis in cases of DIC with the fibrinolytic phenotype is difficult to demonstrate and can be observed only with the use of antifibrinolytic agents because of hyperfibrin(ogen)olysis [2,9,10,24,25].

## Conclusions

The majority of patients with the ACOTS clearly meet the criteria for a diagnosis of DIC with the fibrinolytic phenotype, including very similar sequential changes observed in most of the relevant hemostatic markers. This observation supports our hypothesis that ACOTS can be considered the same disease entity as DIC of the fibrinolytic phenotype. Furthermore, the lower antithrombin levels observed in patients with ACOTS and DIC implies a same pathophysiology as well as same treatment indications for trauma patients with these two different concepts of coagulopathy, which also supports our hypothesis. Most relevantly, the JAAM DIC score obtained in trauma patients immediately after arrival in the emergency department can be used as an independent predictor of higher mortality risk and the need for massive transfusion and damage-control resuscitation

## Key messages

• DIC with the fibrinolytic phenotype exhibits consumption coagulopathy associated with increased fibrin(ogen)olysis and a lower level of antithrombin immediately after arrival in the emergency department and during the early stage of trauma.

• Almost all ACOTS patients meet the criteria for a diagnosis of DIC; therefore, the same changes in the platelet counts, global markers of coagulation and fibrinolysis, and antithrombin levels are observed in patients with DIC and ACOTS.

• The JAAM DIC score obtained immediately after arrival in the emergency department is an independent predictor of the need for a massive transfusion and of death in trauma patients.

## Abbreviations

ACOTS: Acute coagulopathy of trauma-shock; APACHE II: Acute Physiology and Chronic Health Evaluation II; CI: confidence interval; DIC: disseminated intravascular coagulation; FDP: fibrin fibrinogen degradation products; FFP: fresh frozen plasma; ISS: Injury Severity Score; ISTH: International Society on Haemostasis and Thrombosis; JAAM: Japanese Association for Acute Medicine; MODS: multiple organ dysfunction syndrome; PRBCs: packed red blood cells; SIRS: systemic inflammatory response syndrome; SOFA: Sequential Organ Failure Assessment.

## Competing interests

The authors declare that they have no competing interests.

## Authors’ contributions

SG, IT, and HM participated in determining the study design and interpreting the data, and helped to draft the manuscript. AO, YY, and NH collected and assessed the data. All authors read and approved the final version of the manuscript. Each author participated sufficiently in the work to complete this manuscript.

## Supplementary Material

Additional file 1 **The details of the number of patients in Figures** 2** and **3**.** Click here for file
